# Hurdles at work: perceptions of hospital food handlers

**DOI:** 10.1186/1478-4491-7-63

**Published:** 2009-07-24

**Authors:** Cilce Helena Figueiredo Preza Bertin, Magda Andrade Rezende, Dirce Maria Sigulem, Tania Beninga Morais

**Affiliations:** 1 Department of Food and Nutrition, Federal University of Mato Grosso, Cuiabá, MT, Brazil; 2 Department of Mother and Child Health and Psychiatric Nursing, School of Nursing, University of Sao Paulo, São Paulo, SP, Brazil; 3 Postgraduate Program in Nutrition, Federal University of Sao Paulo, São Paulo, SP, Brazil; 4 Food Quality Control Laboratory, Federal University of Sao Paulo, São Paulo, SP, Brazil

## Abstract

**Background:**

Food handlers have a very important role in preventing food contamination during its preparation and distribution. This responsibility is even greater in hospitals, since a large number of patients have low immunity and consequently food contamination by pathogenic bacteria could be particularly harmful. Therefore, a good working environment and periodic training should be provided to food handlers by upper management.

**Methods:**

This study is qualitative research by means of focus group and thematic content analysis methodologies to examine, in detail, the statements by food handlers working in the milk and specific-diet kitchens in a hospital to understand the problems they face in the workplace.

**Results:**

We found that food handlers are aware of the role they play in restoring patients' health; they consider it important to offer a good-quality diet. However, according to their perceptions, a number of difficulties prevent them from reaching this aim. These include: upper management not prioritizing human and material resources to the dietetic services when making resource allocation decisions; a perception that upper management considers their work to be of lesser importance; delayed overtime payments; lack of periodic training; managers lacking administrative skills; insufficient dietitian staff assistants, leading to overwork, at the same time as there is an excess of dietitians; unhealthy environmental working conditions – high temperature, high humidity, loud and constant noise level, poor ventilation; lack of food, and kitchen utensils and equipment; and relationship conflicts with chief dieticians and co-workers.

**Conclusion:**

From these findings, improvement in staff motivation could be achieved by considering non-financial incentives, such as improvement in working conditions and showing appreciation and respect through supervision, training and performance appraisal. Management action, such as investments in intermediary management so that managers have the capacity to provide supportive supervision, as well as better use of performance appraisal and access to training, may help overcome the identified problems.

## Background

According to the *Codex alimentarius *[1], a food handler is defined as "any person who directly handles packaged or unpackaged food, food equipment and utensils, or food contact surfaces and is therefore expected to comply with food hygiene requirements". Thus, food handlers have a very important role in preventing contamination during food preparation and distribution. This responsibility is even greater in hospitals, since a large number of patients have low immunity and consequently food contamination by pathogenic bacteria could be particularly harmful [2]. Therefore, a good working environment and periodic training should be provided by upper management to food handlers [3].

Certain characteristics concerning these professionals, such as poor educational level, low socioeconomic level, rapid staff turnover, literacy and language problems as well as poor motivation due to low pay and job status, can contribute to poor professional performance at work and lack of impact of training initiatives [4]. In this context, qualitative research provides a sensible tool to understand a phenomenon in its context, by studying people in their own settings and interacting with them on their own terms [5].

This method allows for the collection of descriptive data via direct and interactive contact of the researcher with the object of the study, obtaining information where the meaning of something or of a situation is the essential topic of interest. This kind of research allows for an understanding of the dynamics of social relations, and also the comprehension of the structures and institutions as a result of human actions, seeking to depict the participant's point of view [6,7].

Within the qualitative approach, focus groups represent a useful way to obtain data that provide detailed descriptions of experiences and beliefs regarding a particular topic of interest to the researcher. With the help of predetermined guidelines, free discussion is stimulated, starting with general issues then moving on to more specific issues. The subjects within a focus group should be homogeneous with respect to their social roles and categories [6,7]. This homogeneity allows for the participants' interaction and discussion of their opinions together with the views and perspectives of the other participants [6,7].

One of the methods used to carry out the data analysis of a qualitative study is thematic content analysis. This is a research tool used to determine the presence of certain words or concepts within texts or sets of texts, to understand the contents of the messages through both quantitative and qualitative indicators. The contents of the texts are transcribed, and the frequency of the emerging themes, the importance and meaning that the research subjects attribute to them, and the relationships among concepts are examined [8].

A previous study [2] showed that food contamination had occurred in Brazilian hospitals, raising awareness of this issue and how important it is to have well-trained food handlers. The objective of this article was to investigate the perceptions of hospital food handlers to understand the problems they face in their workplace. Milk and specific-diet kitchen workers were chosen because they prepare the food served to the most vulnerable patients in the hospital.

## Methodology

### Participants

The participants in this study were food handlers at a public, tertiary teaching hospital with 743 beds (651 general and 92 paediatric) in Brazil. The dietetic service of the hospital had 240 employees, 19 of whom worked in the milk and specific-diet kitchens. Of these, 15 participated in the study. They were responsible for the preparation and distribution of the diets.

For socioeconomic characterization of the participants, participants completed a questionnaire comprising questions on age, gender, level of education, organization tenure, number of the years within the organization and number of the years in the current position. The participants were guaranteed that any and all information obtained during the interviews would be confidential and that participation was voluntary. They each gave written authorization for the recording of the interviews.

The human resources department of the hospital backed the study on the understanding that its results would provide insight for corrective action. The present study was approved by the Committee of Ethics in Research of the Federal University of São Paulo.

## Methods

### In-depth observational study of the daily routine of food handlers

Prior to establishment of the focus group, one of the researchers (CHFPB) carried out an in-depth observational study of the daily routine of food handlers. Findings were used later to understand and analyse the data obtained from the focus group.

### Focus groups

A pilot test was conducted as a pre-test of the interview guide to check on topics of interest, focusing on job difficulties; strategies to deal with the said difficulties; relationships with co-workers and superiors; and the participants' perception of the importance of training.

An experienced and skillful moderator was hired to conduct the focus group sessions. The participants were divided into two groups, one with nine participants and other with six participants. The sessions were held on four different days, two days for each group. Each session lasted 90 minutes on average and all sessions were audiotaped by means of two portable tape recorders. Six hours of recording were integrally and literally transcribed [6].

### Thematic content analysis

The research tool of thematic content analysis was used to determine the presence of certain words or concepts within texts or sets of texts to understand the contents of the messages through both quantitative and qualitative indicators. The contents of the texts are transcribed, and the frequency of the emerging themes, the importance and meaning that the research subjects attribute to them and the relationships among concepts are examined [8].

The transcriptions were coded, or broken down into manageable categories on a variety of levels – words, word sense, phrases, sentences or themes – focusing on and coding for specific words or patterns that were indicative of the research question: the difficulties that the subjects faced at work. The data were classified and grouped into three categories: (1) upper management performance; (2) dietetic service staff members' performance; and (3) training policy. A category is a group of words or themes with similar meanings or connotations [8].

## Results and Discussion

All participants were adult women with a mean age of 50 years (minimum age: 35; maximum age: 58). They had a low educational level, with an average of only eight years of schooling (ranging from four to eleven years), corresponding to Brazilian primary education. The participants worked 12-hour shifts, with 36 hours off-duty, thus completing a 48-hour week. Unlike the situation in other food service establishments, turnover was not a problem, probably because most of the food handlers (11/15) were public servants with secure job tenure. They were experienced professionals with an average of 15 years in their present position, their experience having been acquired while working in the present hospital.

### Content analysis – quotes

The findings of the three categories – upper management performance, dietetic service staff members' performance and training policy – are summarized in Table 1. Comments on each topic are described below. The perceptions were not influenced either by the participants' age or by their length of professional experience, probably because these were homogeneous characteristics. The views presented were held by the majority of participants. It must be strongly emphasized that the views presented are the views of the participants. Managers' views were not investigated in this study.

**Table 1:** Key findings from focus group discussions

**Categories**	**Themes**
**Upper-management performance**	Upper management considered their work to be of lesser importance Upper management did not prioritize human and material resources to the dietetic services when making resource allocation decisions Delayed overtime payments Unhealthy environmental working conditions Lack of food, kitchen utensils and equipment Lack of training policy

**Dietetic service staff members' performance**	1. Lack of leadership skills Organizational structure was strictly hierarchical Power was given to persons only according to their position and technical background Clear separation between the professionals managing and supervising the services and those executing them Questions related to interpersonal interactions were emphasized, quite often involving conflict between supervisors and subordinates Complaints about bad and disrespectful treatment by superiors Superiors focused on finding faults instead of solving problems Meetings for discussing problems were seldom held; were scheduled at times when not all the employees were available to participate; seemed to be to solve delicate and personal questions; on some occasions, strangers to the department were present Superiors were not fair, since they often blame the employees for mistakes they had not committed 2. Lack of ability to build effective teamwork Some co-workers managed to secure favoritism by the superiors Some co-workers with public servant status had disrespectful and defiant attitudes towards their superiors, somehow inhibiting the superiors from taking stronger measures, so that those who were more obedient and respectful to orders ended up overloaded Some co-workers with public servant status had low commitment to the job 3. Lack of clear objectives, job descriptions and tasks Inadequate distribution of personnel and tasks among the departments Excessive workload because of a shortage of personnel Superiors did not give staff a clear vision of the department's goals Information about working conditions, tools, equipment used, knowledge and skills needed, and relationships with other positions was not well understood Too many people in charge, making it difficult to know to whom to communicate in the chain of command No satisfatory pay or benefits

**Training policy**	Lack of periodic training as a demonstration that they were not of sufficient importance to deserve training courses Initial training for new co-workers placed under the responsibility of older and more experienced colleagues Concern that it would be difficult to apply proper food handling techniques learnt on training courses, given the precarious working conditions

### Upper management performance

According to the participants, upper management, when allocating financial resources, prioritized the hospital's other services to the detriment of the dietetic service, which resulted in resentment. The participants were also critical of how public funding was spent. There were no clear priorities, and scarce resources were used in situations they considered to be of lesser importance, an example being Christmas parties.

The participants main complaint was delay in overtime payment. Often they felt it necessary to personally put pressure on the administrator to obtain their overtime pay. This fact was repeatedly raised and caused the participants embarrassment and humiliation. For the participants, the fact that the administration did not honor its obligation discredited the institution in their eyes and made them wary of working further overtime:

"They pay the security guards. Why they don't pay us?" (milk kitchen worker)

"They have money for barbecue parties, but not to pay us?" (milk kitchen worker)

Nevertheless, a sense of obligation toward co-workers and patients led them to grant the chief dietitians' requests to do replacement and overtime work. They felt that if they did not, co-workers would be overwhelmed and, consequently, jeopardize patients' health care:

"I have told them [the managers]: I don't do this [work overtime] for you. I do it for [the sake of] the children [paediatric patients]." (milk kitchen worker)

" [Agreeing] And, also, to help the colleagues." (specific-diet kitchen worker)

Unhealthy environmental working conditions – high temperature, high humidity, loud and constant noise level and poor ventilation – were also mentioned as negative factors affecting the work performance of the participants, particularly in the milk kitchen. They emphasized that they worked in a confined environment, in a very small room, with extremes of temperature and, even worse, in a standing position most of the time. Also, many people working within a restricted area hampered movement, creating a stressful situation. For the study's participants, these conditions increased the probability of errors because they provoked irritation and mental and physical fatigue. They were also perceived to have harmful effects on their health:

"Water baths, autoclaves and stoves, all generating heat and [the room has] no air conditioning." (milk kitchen worker)

" [At the end of the workday] I feel very, very tired and like as leaving a sauna." (specific-diet kitchen worker)

"Conveyor belt, pan lids, shouts: 'Take this, take that', an incredible noise." (specific-diet kitchen worker)

The participants working in the specific-diet kitchen felt isolated and forgotten because the kitchen was located in the hospital basement, separated from other departments, contributing to the feeling that they were different from the other employees:

"We are hidden, invisible to the world." (specific-diet kitchen worker)

Adding to this already precarious situation, the participants referred to the lack of utensils and equipment, and even of food, forcing them to use incorrect procedures, of which they were fully aware. Participants often bought or brought their own utensils from home. Frequently, equipment went out of service due to the lack of maintenance, thereby causing extra work, delays in meal delivery and even having to redo the work. In the face of such a situation, the participants had to exercise creativity and improvisation:

"A few days ago, the stove was not working. I had to cook the vegetables [for the paediatric patients' soup] in the autoclave!" (specific-diet kitchen worker)

A particularly disturbing situation was the shortage of food supplies. Besides this, often the food available was of poor quality. The participants stated that there were frequently last-minute changes to the menu, with consequent disruption in the execution of their daily tasks, resulting in double the work. On some occasions, they themselves resolved the problem by buying or bringing food from their own homes. Sometimes the problem was so critical that the meals were prepared using only water and oil, making them tasteless. According to the participants' opinion, superiors and patients unfairly blame them for the poor quality of the meals:

"How can we make tasty meals if the food quality is not good?" (specific-diet kitchen worker)

"We are scapegoats [for the bad quality of the food]." (specific-diet kitchen worker)

They would have liked to offer good-quality meals to the patients, but this was not possible because of these constraining factors. Consequently, feelings of anxiety and helplessness were common, contributing to low self-esteem. Despite these facts, their actions demonstrated clear solidarity toward the patients, with special regard for paediatric patients.

For the participants working in the milk kitchen, the issue of feeding-bottle contamination was also an additional factor contributing to anxiety, because they were responsible for the making up of sound feeding bottles:

"I keep worrying ... What did I do wrong? (milk kitchen worker)

"I don't even want to talk ... [about this issue]." (milk kitchen worker)

To put these findings into the broader context, it is necessary to stress that the annual health expenditure was only USD 85.90 per capita in 2002 in Brazil. In 2004, the deficit in the health budget was estimated at USD 600 000 [9], resulting in low funding for the hospital's services, delays in reimbursements and bad quality of service.

### Dietetic service staff members' performance

Managerial skills were widely seen as the main factor influencing the success of job performance. The participants stressed that their superiors did not have the necessary administrative skills required for food service managers. This situation led to dysfunctional interaction with subordinates. According to the participants, the lack of leadership skills and the lack of job and task descriptions were the main reasons for their frustration.

#### Lack of leadership skills

According to statements of the participants, the organization had a strictly hierarchical structure, where formal power was given to persons only according to their position and technical background. There was a very clear separation between the professionals managing and supervising the services and those executing them. Within this top-down management style it was expected that those holding the upper hierarchical positions have the ability to think, make decisions and give orders on how to proceed in each case.

The importance of building effective interpersonal communication was strongly stressed. According to the statements of the participants, there was no space for open and sincere dialogue between the staff members and their superiors, making the workplace atmosphere tense. Questions related to interpersonal interactions were emphasized, quite often involving conflict between supervisors and subordinates. Complaints were frequent about ill and disrespectful treatment by superiors. According to the participants, this low standard of employee treatment led to feelings of dissatisfaction and resentment among them. This is more evident in the specific-diets kitchen, where little affinity between employees and supervisors was evident. The milk kitchen employees did not face this problem:

"It seems that she [the supervisor] doesn't know those two little magical words: 'please' and 'thank you'." (specific-diet kitchen worker)

It is noteworthy that, in addition to harsh words about their superiors, the participants also criticized some of their co-workers. According to them, some colleagues managed to benefit from favoritism by the superiors, obtaining some sort of advantage at work.

Other co-workers had disrespectful and defiant attitudes towards theirs superiors. The participants believed that these attitudes somehow inhibited the superiors from taking stronger measures when necessary, such that those who were more obedient and respectful to orders ended up overloaded with work. They considered the public servant status a contributing factor to this behaviour, because their status gave public servants the security of job tenure. This situation, where two kinds of labour relations coexisted, led to tension among the employees. The participants also reported that some co-workers did not get along well:

"When asked to do something [by the supervisors], they [the public servants with job tenure] say they won't." (specific-diet kitchen worker)

"That's because they know they won't lose their jobs." (specific-diet kitchen worker)

According to the participants, superiors focused on finding faults instead of solving problems. Meetings for discussing problems were seldom held. When they did happen, they were scheduled at times when not all the employees were available to participate. Whenever these meetings did take place, it seemed that the superiors wanted to solve delicate and personal questions. To the participants, this practice seemed inappropriate. A more serious situation was that on some occasions, strangers to the department were present. In addition, the participants felt that their superiors were not fair, since they would often blame them for mistakes they had not committed:

"Meetings are [held] only for the sake of complaints." (specific-diet kitchen worker)

#### Lack of clear objectives, job descriptions and task lists

For the participants, the superiors did not give staff a clear vision of the department's goals. For them, participating in setting objectives would give meaning and purpose to the job. Understanding how their job fit the big picture would help them to properly develop their functions, to feel important and be involved with their tasks. Lack of job and task descriptions added to workplace confusion, hurt communication and contributed to staff members' not knowing what was expected of them. Information about working conditions, tools, equipment used, knowledge and skills needed and relationships with other positions was not well understood. For them, there were many people in charge, making it difficult to know to whom to communicate within the chain of command. Inadequate distribution of personnel among the sectors was also reported. The participants called the situation "lack of organization". Most of them felt unsatisfied with pay and benefits. Also, they thought that others co-workers were better paid for the same tasks.

The milk kitchen seemed to have specific problems. The workload was considered excessive because of a shortage of personnel. Moreover, staff members felt that the superiors were more demanding of them, compared to other employees. With regard to vacations, sick leave and days off, replacement of personnel was difficult because they had very specific skills, such that other employees could not fill in for them, resulting in an even heavier workload for those remaining:

"A lot of work for just four people." (milk kitchen worker)

"Seven straight working hours. We don't stop a minute." (milk kitchen worker)

When questioned about the recognition of their work by the superiors, there were mixed feelings: some of the participants believed they were recognized, while others felt their contribution was not valued and that they were only "necessary" to get the job done. Participants worked for a variety of reasons: to earn a living; for personal fulfillment; to contribute to something important; and to feel that they were helping patients through their work. They also liked the camaraderie and interaction with co-workers. Recognition and praise from superiors were greatly appreciated. For them, a working environment in which people feel important and appreciated would contribute to better job satisfaction.

Interestingly, the participants acknowledged that the situation also affected their superiors negatively. The participants felt that the superiors were not motivated because of the job security provided by their tenure. Entrenched attitudes such as "We don't get paid extra to work harder" and "I'm going to do as little as possible" were seen in some superiors.

#### Lack of training policy

The participants showed an accurate perception of the meaning of training. For them, training would update knowledge, developing skills and improving their ability to perform their tasks. However, the fact that no regular training programmes were in place was perceived by them as a sign that their superiors believed they were not of sufficient importance to deserve training courses. Initial training for new co-workers was placed under the responsibility of older and more experienced colleagues. The participants also expressed concern that given the precarious working conditions, it would be difficult to apply any proper food handling techniques learnt on training courses:

"The last [training] happened many years ago. We are forgotten." (milk kitchen worker)

## Conclusion

This was a cross-sectional, qualitative, research study on the perceptions of food handlers in a public hospital located in a developing country, Brazil. However, a number of findings may be generalized to public hospitals in other countries. As with other health workers [10], good working conditions and appreciation by managers, colleagues and patients are considered important for job motivation. Moreover, as others food handlers, they also faced barriers such as lack of time, staff and resources [11].

The results showed that hospital food handlers were aware of the role they play in helping patients recover their health: offering a good-quality diet was considered by them to be very important. However, according to them, a number of difficulties such as unaware or absent superiors, inadequate working conditions, outdated or ill-functioning equipment, lack of recognition and lack of training prevent them from performing optimally.

From these findings, the improvement of staff motivation could be achieved through non-financial incentives, such as improvement of working conditions and showing appreciation and respect through supervision, training and performance appraisal. Management action, such as investment in intermediary management so that managers have the capacity to provide supportive supervision, better use of performance appraisal and access to training, may help to overcome the identified problems. However, improved human resources management alone cannot compensate for the lack of investment and the structural deficits that characterize health systems in many developing countries.

## Competing interests

The authors declare that they have no competing interests.

## Authors' contributions

CB participated in conception and design of the study; in acquisition, analysis and interpretation of data and in drafting the manuscript. MR participated in conception and design of the study, in analysis and interpretation of data and in drafting the manuscript. DS participated in conception and design of the study. TM participated in conception and design of the study, in revising the manuscript and by giving final approval of the version to be published. All authors read and approved the final manuscript.
